# Lower Extremity Amputations in Persons with and without Diabetes in Italy: 2001–2010

**DOI:** 10.1371/journal.pone.0086405

**Published:** 2014-01-28

**Authors:** Flavia L. Lombardo, Marina Maggini, Alessandra De Bellis, Giuseppe Seghieri, Roberto Anichini

**Affiliations:** 1 National Centre for Epidemiology, Surveillance and Health Promotion, National Institute of Health, Roma, Italy; 2 Diabetes Unit and Diabetic Foot Unit, Department of Internal Medicine General Hospital Pistoia, Pistoia, Italy; German Diabetes Center, Leibniz Center for Diabetes Research at Heinrich Heine University Duesseldorf, Germany

## Abstract

**Objective:**

To analyze hospitalization for lower extremity amputations (LEAs) and amputee rates in persons with and without diabetes in Italy.

**Research Design and Methods:**

All patients with LEAs in the period 2001–2010 were identified analyzing the National Hospital Discharge Record database. For each year, amputee and hospitalization rates for LEAs were calculated either for persons with diabetes or without. Time trend for major and minor amputations were analysed.

**Results:**

From 2001 to 2010 a mean annual number of 11,639 individuals underwent a lower extremity amputation: 58.6% had diabetes accounting for 60.7% of total hospitalizations. In 2010, the crude amputee rate for LEAs was 20.4 per 100,000 inhabitants: 247.2 for 100.000 persons with diabetes, and 8.6 for those without diabetes. Having diabetes was associated to an increased risk of amputation (Poisson estimated RR 10.9, 95%CI 9.4–12.8). Over the whole period, a progressive reduction of amputee rates was observed for major amputations either among persons with diabetes (−30.7%) or without diabetes (−12.5%), while the rates of minor amputations increased progressively (+22.4%) among people without diabetes and were nearly stable in people with diabetes (−4.6%). A greater number of minor amputations were performed among persons with than without diabetes: in 2010, the minor-to-major ratio among persons with diabetes (2.5) was more than twice than in those without diabetes (1.0).

**Conclusions:**

The nationwide analyses confirm a progressive reduction of hospitalization and amputee rates for major LEAs, suggesting an earlier and more diffuse approach aimed at limb salvage.

## Introduction

As in other European countries the prevalence of known diabetes in Italy is constantly increasing. Based on the Italian National Institute of Statistics (ISTAT) estimates in the year 2012, there were more than 3,000,000 persons with diabetes constituting 5.5% of the population (5.5% of females and 5.4% of males). The age-sex standardized prevalence has been increasing from 3.9% in 2001 to 5.0% in 2012. Prevalence increased with age, reaching 20% in persons older than 75 years. As to territorial distribution, prevalence of diabetes has a South-North gradient being the highest in the South (6.2%), followed by Centre (5.5%) and North (4.9%) of Italy (www.epicentro.iss.it/igea/en/FactSheet.asp).

In 2005 the International Diabetes Federation (IDF) chose to focus on one of the most severe complications of diabetes, such as diabetic foot disease. Personal, social and economic costs and the incidence of lower extremity amputations (LEAs) in diabetes increase with age so that the number of diabetes-related amputations can be expected to increase in the next years. Saint Vincent Declaration, dating back to 1989, and aimed at reducing LEAs by 50% within 5 years [1], [2] is far from being implemented. Moreover, although amputations are considered a ‘negative’ quality marker of foot care in diabetes [3], available data on diabetic foot problems and amputation rates show strong variation among different populations in Europe [4]–[11], further demonstrating that a standardized approach to this problem is indeed lacking. In Europe the main trend is the reduction in major amputation rates and in some countries, the observed improvement in amputation incidence could be related to the implementation of the International Consensus of the Diabetic Foot (ICDF) [10] or as a benefit of multidisciplinary team work [8], [9] while in other countries such as the Netherlands, to the increase in number of podiatric services [11]. The issue is however open since an accurate study concerning both major and minor amputations has not, till now, been accomplished.

In Italy, few data have been so far available about lower limb amputations in persons with diabetes, and no nationwide study attempted to evaluate the time trend of this important complication. Aim of this study was to analyze amputee and hospitalization rates for LEAs in persons with and without diabetes, using the National Hospital Discharge Record database for the period 2001–2010.

## Materials and Methods

### Data source

Data were extracted from the National Hospital Discharge Record database held by the Italian Ministry of Health (http://www.archeo.salute.gov.it/ricoveriOspedalieri/ricoveriOspedalieri.jsp). This database contains administrative and clinical data regarding all yearly admissions to public and private hospitals in Italy, including day-hospital (less than 24-hour of stay). For each hospitalization, information available from database and used in the present study is: hospital code, individual patient code, age, sex, region of residence, citizenship, admission and discharge dates, discharge status (i.e. ordinary, voluntary discharge, or transferred to other hospital, dead), main discharge diagnosis and up to five additional diagnoses, Diagnosis Related Group (DRG) code, main procedure as well as up to five further procedures. Diagnoses and procedures are coded using the International Classification of Diseases-Clinical Modification, 9^th^ edition (ICD9-CM). Data provided by the Ministry of Health are anonymous but the individual code allows for the record linkage on the same patient over different years.

All hospital discharge records, from January 1^st^ 2001 to December 31^st^ 2010, with lower extremity amputations (ICD9 84.10–84.19) in primary or in one of the five secondary procedures were identified. Duplicated records (same admission and discharge date for the same patient) were excluded. Moreover, patients were excluded if aged more than 100 years, and/or had traumatic (ICD-9-CM 895–897; DRG 442–443) or tumour-related peripheral amputations (ICD-9-CM 170.7, 170.8; DRG 213, 408). The resulting database consisted of 135,805 records.

Census data, as well as information on diabetes prevalence by year, age, sex, and geographical area were obtained from ISTAT (www.istat.it). Neither ethical approval nor individual written consent by patients were requested as the data were retrospective and anonymous.

### Definitions

For each year, a person with diabetes with LEA was defined as a person who had a hospital discharge with LEA, and diagnoses of diabetes (ICD9-CM: 250.xx in any field) on the same date or in any discharge occurring in the same year. All the LEA hospitalizations, referred to a person with diabetes, occurring in the same year were considered diabetes-related. Two or more amputations occurring during the same hospitalization were considered as a single event because it was not possible to assess whether amputations were bilateral or contralateral, and if the second one was a review of the previous one.

Lower limb amputations were classified according to their level: a minor amputation refers to any LEA below the ankle joint (ICD9-CM: 84.11–84.12); a major amputation refers to any LEA above the ankle joint (ICD9-CM: 84.13–84.19). Amputations without the level specification (ICD9-CM: 84.10) were considered only in the overall rates.

### Under-reporting of diabetes diagnosis

In order to estimate possible under-reporting of diabetes diagnosis in amputated patients, a control was performed as follows: all amputees without diabetes diagnosis in the year 2010 were identified; for each identified patient, any discharge with a diagnosis of diabetes in the preceding years (2001–2009) was searched in the hospital discharge database.

### Analysis

For each year, the amputee rate was calculated as the ratio of the number of patients discharged with amputation to the Italian resident population with and without diabetes; thus, if a patient underwent more than one LEA in the same year, he/she was included only once according to the most severe episode (i.e. the highest level). Persons with diabetes were calculated from ISTAT diabetes prevalence estimates. The population without diabetes was calculated by subtracting the estimated population with diabetes from the total Italian population for each year. The hospitalization rate for LEAs was calculated as the ratio of the number of discharges with LEAs to the Italian resident population with and without diabetes.

Hospitalization and amputee rates were standardized using the 2001 total Italian resident population (ISTAT). Direct method was used to standardize by sex and age grouped in seven classes: 0–34, 35–44, 45–54, 55–59, 60–64, 65–74, 75 and over.

Because of the lack of individual codes for some Italian regions in 2001 and 2002, amputee rates were calculated only for the years 2003–2010. For the same reason, 2001–2002 hospitalization rates of diabetes-related LEAs could be underestimated.

To test the time trend, the Poisson regression model was used, separately for persons with and without diabetes; calendar year, sex and age categorized in seven classes were considered in the model as independent variables. To compare rates in persons with and without diabetes, the Poisson model was fitted to the entire population including a variable for diabetes status, and an interaction term was also included to determine if the rate ratio changed over the study period. The analyses using Poisson model were done for the total of amputations, and also considering only minor and major LEAs respectively.

Statistical analyses were performed using STATA version 11.0 (STATA corp LP, College Station TX USA).

## Results

During the period 2001–2010, a mean annual number of 11,639 individuals underwent a lower extremity amputation, among them 58.6% had diabetes accounting for 60.7% of the total number of hospitalizations (mean annual number 13,581).

**Figure 1:**
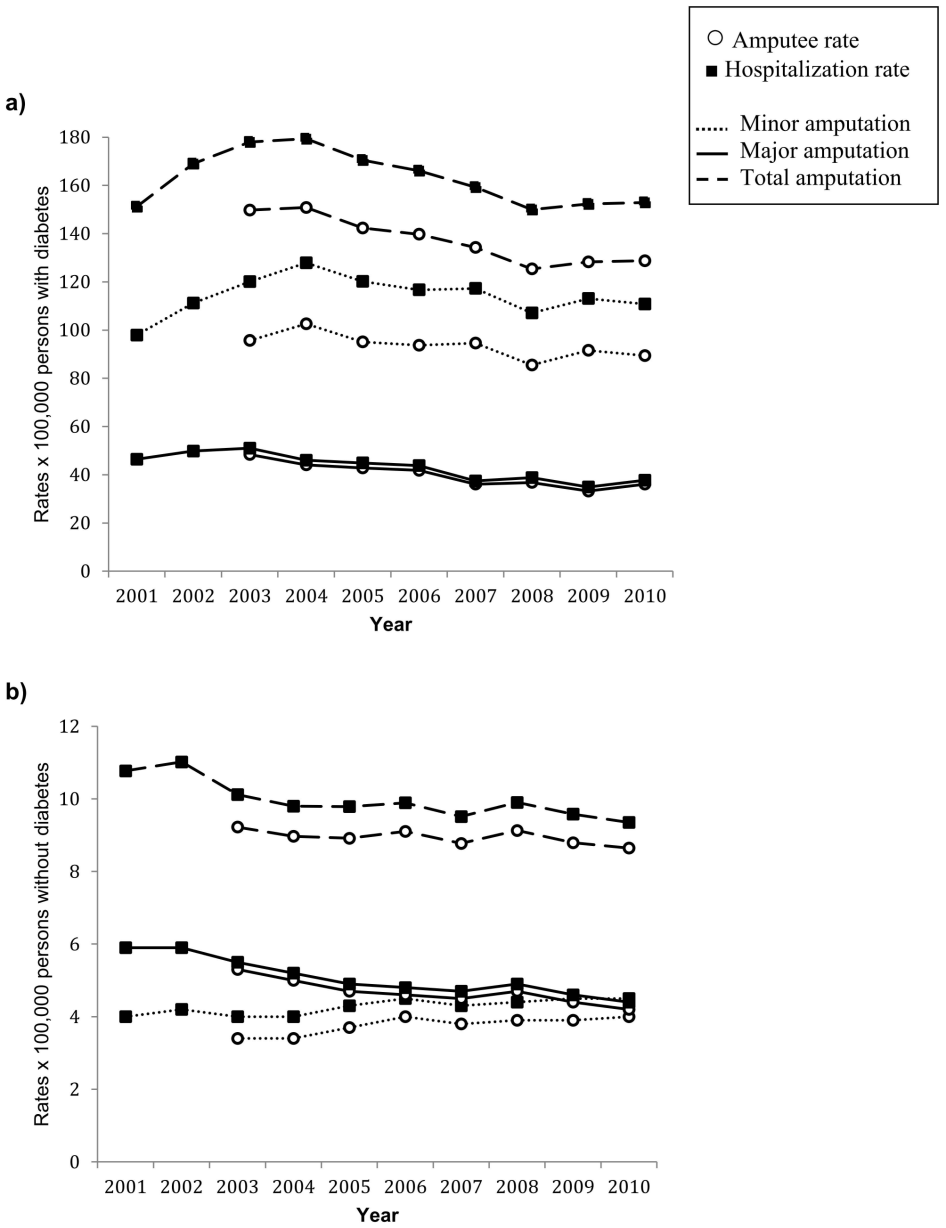
Age and sex standardized amputee and hospitalization rates in persons (a) with and (b) without diabetes. Italy, 2001–2010.

The crude amputee rate for LEAs in the year 2010 was 20.4 per 100,000 inhabitants: 247.2 for 100,000 persons with diabetes, and 8.6 for those without diabetes (Table 1), the male-to-female ratio was 2.7 among individuals with diabetes and 1.6 among those without diabetes. Persons with diabetes underwent amputation procedures at a younger age with respects to persons without diabetes (71.1±11.1 vs. 73.2±17.9 years). Considering the discharges with LEAs, the crude hospitalization rate was 23.5 per 100,000 inhabitants: 297.0 for persons with diabetes, and 9.3 for those without diabetes (Table 1). A greater number of minor amputations were performed among persons with than without diabetes: the minor-to-major ratio among persons with diabetes (2.5) was more than twice than in those without diabetes (1.0). Table 2–3 shows the number of amputees and hospitalizations for each year either for persons with diabetes or without. In addition, crude and standardized rates (standard: Italian population) are presented for all, minor and major amputations. In the study period, a progressive reduction of amputee rates was observed for major amputations either among persons with diabetes (−30.7%) or without diabetes (−12.5%); the rates of minor amputations were nearly stable in people with diabetes (−4.6%), and increased progressively (+22.4%) among people without diabetes (Table 2–3, Figure 1). The Poisson model confirmed the observed trends: for major LEAs, the model estimated a rate ratio (RR) per year of 0.95 (95%CI 0.94–0.97, p<0.001) for persons with diabetes and 0.98 (95%CI 0.95–0.99, p<0.001) for persons without diabetes; for minor LEAs the RR was 1.0 (95%CI 0.99–1.01, p = 0.308) and 1.02 (95%CI 1.01–1.03, p<0.01) respectively. Similar trends were observed for hospitalization rates.

**Table 1:** Rate of amputees (top) and hospitalization for amputation (bottom) in persons with and without diabetes, Italy 2010.

	With diabetes	Without diabetes
**Amputees**		
Number	7,373	4,922
Crude amputee rate (per 100,000)∧	247.2	8.6
Male to female rate ratio	2.7	1.6
Age* (mean±sd)	71.1±11.1	73.2±17.9
**Hospitalizations for LEAs**		
Number§	8,857	5,339
Crude hosp. rate (per 100,000)∧	297.0	9.3
Minor to major ratio	2.5	1.0

§ All discharges for amputation (i.e. major, minor and not specified level).

∧ Rates are expressed per 100,000 000 persons with and without diabetes respectively.

* Age at first amputation.

**Table 2:** Amputee rate in people with diabetes (rates per 100,000 persons with diabetes) and without diabetes (rates per 100,000 persons without diabetes).

	AMPUTEES											
	People with diabetes						People without diabetes					
	Minor		Major		Total		Minor		Major		Total	
	N	Amputee rate	N	Amputee rate	N	Amputee rate	N	Amputee rate	N	Amputee rate	N	Amputee rate
**2001**	3,024	na	2,232	na	5,523	na	1,652	na	2,597	na	4,628	na
**2002**	3,506	na	2,563	na	6,371	na	1,744	na	2,642	na	4,764	na
**2003**	3,752	168.8 (95.7)	2,538	114.5 (48.4)	6,584	296.6 (149.8)	1,784	3.2 (3.4)	2,641	4.8 (5.3)	4,691	8.5 (9.2)
**2004**	4,143	178.0 (102.6)	2,465	105.9 (44.1)	6,860	294.7 (150.8)	1,797	3.2 (3.4)	2,586	4.7 (5.0)	4,667	8.4 (9.0)
**2005**	4,298	177.0 (95.1)	2,464	101.4 (42.8)	7,014	288.7 (142.3)	1,954	3.5 (3.7)	2,467	4.4 (4.7)	4,723	8.4 (8.9)
**2006**	4,351	167.3 (93.7)	2,469	94.9 (41.8)	7,096	272.8 (139.8)	2,164	3.9 (4.0)	2,483	4.4 (4.6)	4,917	8.8 (9.1)
**2007**	4,258	156.9 (94.6)	2,284	84.1 (36.1)	6,788	250.1 (134.3)	2,102	3.7 (3.8)	2,478	4.4 (4.5)	4,849	8.6 (8.8)
**2008**	4,482	155.1 (85.5)	2,517	87.1 (36.8)	7,241	250.6 (125.4)	2,151	3.8 (3.9)	2,608	4.6 (4.7)	5,051	8.9 (9.1)
**2009**	4,765	163.9 (91.6)	2,382	81.9 (33.2)	7,380	253.8 (128.3)	2,191	3.8 (3.9)	2,474	4.3 (4.4)	4,945	8.7 (8.8)
**2010**	4,794	161.1 (89.4)	2,362	79.3 (36.1)	7,373	247.2 (128.7)	2,267	4.0 (4.0)	2,411	4.2 (4.2)	4,922	8.6 (8.6)

Age and sex standardized rates (in brackets) are calculated on the basis of 2001 Italian resident population.

Note. Total amputations: minor, major and not specified level.

**Table 3:** Hospitalization rate for amputation in people with diabetes (rates per 100,000 persons with diabetes) and without diabetes (rates per 100,000 persons without diabetes).

	HOSPITALIZATIONS FOR AMPUTATION											
	People with diabetes						People without diabetes					
	Minor		Major		Total		Minor		Major		Total	
	N	Amputee rate	N	Amputee rate	N	Amputee rate	N	Amputee rate	N	Amputee rate	N	Amputee rate
**2001**	4,038	181.7 (97.9)	2,440	109.8 (46.4)	6,842	307.8 (151.2)	2,046	3.7 (4.0)	2,911	5.3 (5.9)	5,398	9.9 (10.8)
**2002**	4,649	210.3 (111.2)	2,779	125.7 (49.8)	7,820	353.8 (169.0)	2,141	3.9 (4.2)	2,949	5.4 (5.9)	5,523	10.1 (11)
**2003**	4,892	218.8 (120.1)	2,699	120.7 (51.0)	7,955	355.8 (178.0)	2,099	3.8 (4.0)	2,799	5.1 (5.5)	5,203	9.4 (10.1)
**2004**	5,373	229.9 (127.9)	2,593	111.0 (46.0)	8,298	355.1 (179.3)	2,089	3.8 (4.0)	2,737	4.9 (5.2)	5,139	9.3 (9.8)
**2005**	5,527	226.7 (120.2)	2,606	106.9 (44.9)	8,458	346.9 (170.5)	2,279	4.1 (4.3)	2,610	4.7 (4.9)	5,227	9.3 (9.8)
**2006**	5,578	214.1 (116.7)	2,612	100.3 (43.8)	8,538	327.8 (166.1)	2,474	4.4 (4.5)	2,584	4.6 (4.8)	5,362	9.5 (9.9)
**2007**	5,404	198.9 (117.3)	2,400	88.3 (37.4)	8,110	298.5 (159.3)	2,375	4.2 (4.3)	2,591	4.6 (4.7)	5,264	9.3 (9.5)
**2008**	5,739	198.3 (107.1)	2,690	92.9 (38.8)	8,741	302.0 (150.0)	2,452	4.3 (4.4)	2,726	4.8 (4.9)	5,493	9.7 (9.9)
**2009**	6,014	206.5 (113.1)	2,532	86.9 (34.9)	8,842	303.6 (152.3)	2,520	4.4 (4.5)	2,571	4.5 (4.6)	5,396	9.4 (9.6)
**2010**	6,080	203.9 (110.8)	2,495	83.7 (37.7)	8,857	297.0 (153.0)	2,579	4.5 (4.5)	2,492	4.3 (4.4)	5,339	9.3 (9.4)

Note. Total amputations: minor, major and not specified level.

Age and sex standardized rates (in brackets) are calculated on the basis of 2001 Italian resident population.

Having diabetes was associated to an increased risk of amputation. In 2010, the standardized amputee rate was 128.7 (per 100,000) for persons with diabetes and 8.6 (per 100,000) for persons without diabetes. Considering the whole period, the estimated rate was 11 times higher among people with diabetes compared with those without diabetes (RR 10.9, 95%CI 9.4–12.8). The RR was 19.4 (95%CI 16.5–22.8) for minor LEAs, and 6.4 (95%CI 5.6–7.2) for major LEAs (Table 4). No significant change in the rate ratio was observed over the study period either for minor and major LEAs.

**Table 4:** Relative risk of lower extremity amputation in people with diabetes, compared with those without diabetes, adjusted for age, sex and calendar year: results of the Poisson models.

	MINOR			MAJOR			TOTAL		
	RR	(95%CI)	*p*	RR	(95%CI)	*p*	RR	(95%CI)	*p*
Diabetes (yes vs no)	19.37	(16.49–22.77)	*<0.001*	6.36	(5.60–7.23)	*<0.001*	10.95	(9.37–12.81)	*<0.001*
Year*	1.01	(0.99–1.03)	*0.443*	0.96	(0.94–0.98)	*0.001*	0.98	(0.96–1.01)	*0.203*
Sex (M vs F)	2.56	(2.27–2.88)	*<0.001*	2.10	(1.86–2.38)	*<0.001*	2.33	(2.03–2.67)	*<0.001*
Age§									
35–44	1.75	(1.39–2.21)	*<0.001*	3.04	(2.44–3.77)	*<0.001*	2.07	(1.65–2.61)	*<0.001*
45–54	5.01	(3.86–6.51)	*<0.001*	9.82	(7.32–13.19)	*<0.001*	6.50	(4.81–8.78)	*<0.001*
55–59	7.68	(6.34–9.32)	*<0.001*	22.53	(17.63–28.78)	*<0.001*	11.81	(9.40–14.84)	*<0.001*
60–64	9.86	(8.34–11.65)	*<0.001*	35.36	(28.92–43.23)	*<0.001*	16.49	(13.72–19.83)	*<0.001*
65–74	12.15	(10.58–13.96)	*<0.001*	63.52	(55.77–72.34)	*<0.001*	23.88	(21.12–27.01)	*<0.001*
75 and over	15.77	(12.68–19.62)	*<0.001*	163.49	(142.16–188.02)	*<0.001*	43.92	(36.52–52.83)	*<0.001*

* Relative risk per one year increment;

§ reference group 0–34 years.

The supplementary Table S1 shows amputee and hospitalization rates calculated using Italian resident population as denominator for persons with diabetes; these are widely accepted indicators of quality of care and may be useful in comparing the results with those from other countries.

Over the whole study period, males with diabetes had a higher risk to undergo a lower limb amputation than females with diabetes: the Poisson estimated RR was 2.6 (95%CI 2.5–2.8) for minor amputations, and 2.0 (95%CI 1.9–2.2) for major LEAs. From 2001 to 2010, the male to female RR increased from 2.3 to 3.1 for minor amputations, and from 1.8 to 2.6 for major amputations.

**Figure 2:**
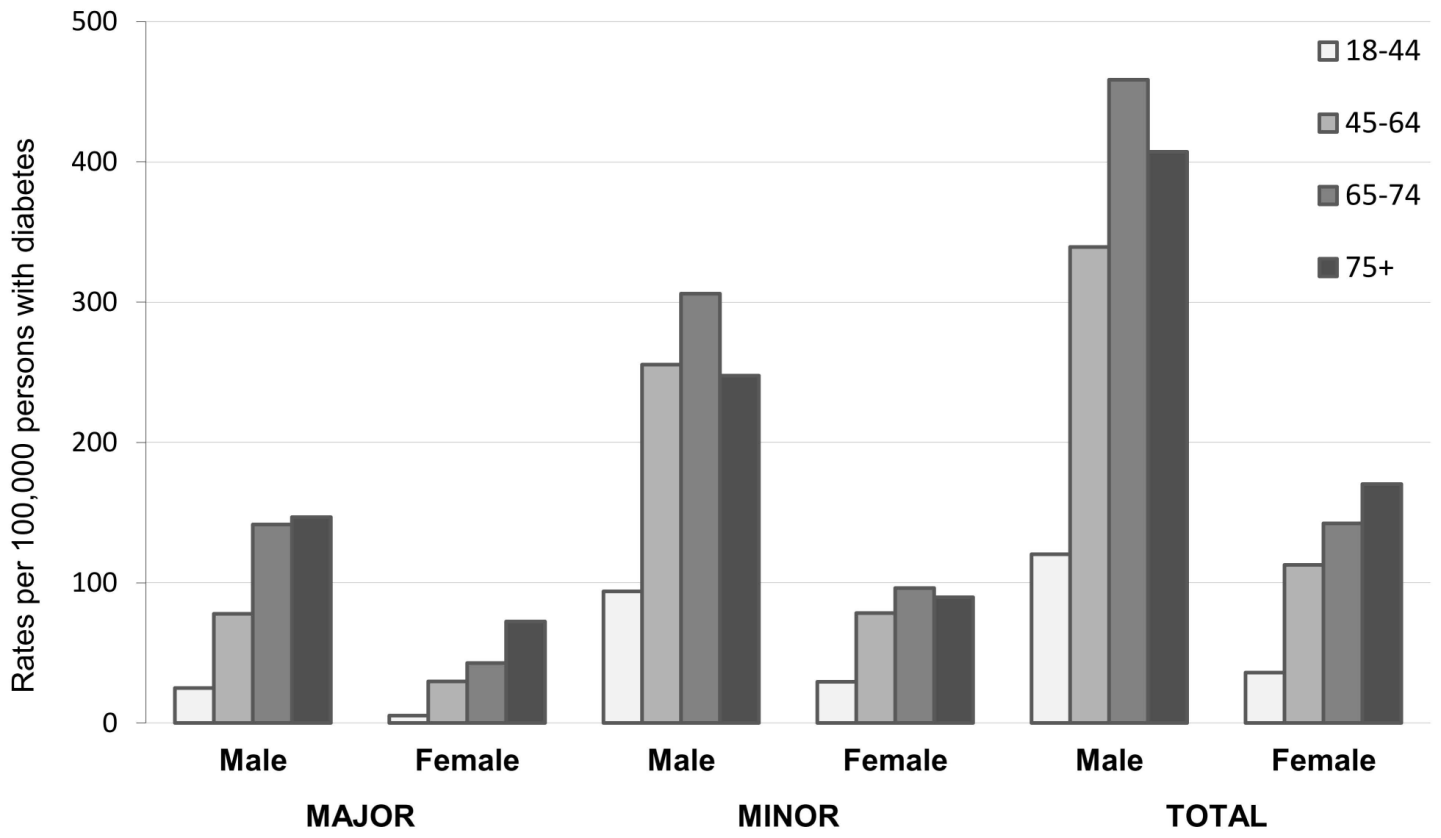
Amputee rate by sex and age classes (major, minor and total amputations) in people with diabetes. Italy, 2010.

Amputee rates strongly increased with age for both males and females with diabetes (Figure 2) in particular for major amputations: comparing persons with more than 75 years of age with those 18–34 year old, RR was 5.9 among males, and 13.6 among females.

### Under-reporting of diabetes diagnosis

A total of 4,922 amputees were identified in the year 2010, without a diagnosis of diabetes. Using the individual patient code, the hospital discharge database was searched for the period 2001–2009: for 805 patients, at least one hospitalization with a diagnosis of diabetes was found. As a result of this procedure, the 885 amputations related to these patients were now considered as diabetes-related. In 2010, the resulting hospitalization rate would be 326.7 per 100,000 persons with diabetes, 10% higher than the rate estimated with the linkage performed only over the same year.

## Discussion

In this paper, we presented data on lower peripheral amputations using two different measures; hospitalization rate was calculated in order to provide a measure of the health care costs, as well as the suffering of the patient, while the amputee rate i.e. the highest LEA, is a better measure of disease severity [12]. Our nationwide analysis confirms what has previously been observed in some European countries and in USA, namely a progressive reduction of hospitalization and amputee rates for major amputations [9], [10], [13]–[19]. The main hypothesis to explain this trend, in agreement with the majority of previously mentioned studies, is based on the fact that, even in presence of raised diabetes incidence and prevalence, we are eventually facing an improvement in quality of diabetes therapy as well as in the overall approach to diabetic foot care (peripheral revascularizations etc.) [20]–[24]. Other studies, on the contrary, have observed a steadiness or an increase in the incidence of amputations among persons with diabetes [4], [6], [25]. To explain such discrepancies it is to note that many studies do not differentiate major from minor amputations, computing both in an unique group [26], [27], and some confounding factors such as age, sex, increased rate of diabetes, together with a difference in severity of disease at presentation, must be taken into account since all these variables may modify this finding [28].

Interestingly, in our study, while the declining trend of major amputation rates was similar either for persons with or without diabetes (Table 2–3, Figure 1), a different picture was observed when minor amputations were considered: an upward trend was observed among individuals without diabetes (+22%) with respect to essentially stable rates among those with diabetes. These diverging trends can be expected, in particular for diabetic patients, since minor amputations are increasingly used in usual routine treatment of diabetic foot, aimed at limb salvage.

Even if European studies concerning minor amputations on national level are relatively scarce, all reports demonstrate discrepancies in minor amputation rates for diabetic foot throughout Europe. This probably reflects differences in severity of foot lesions at presentation as well as in access to specialized foot care among studied populations [28]. With regard to Italy, our data confirm a high rate of minor amputation in agreement with what previously observed by Faglia et al. as well as by a precedent regional study [10], [22]. Reasons for Italian high rate of minor-to major amputations may be due, on one hand to an early approach in revascularization procedures [22]–[24], [29], [30] or, on the other, to a more careful implementation of guidelines concerning prevention and early detection of foot lesions, as observed in Italy in the last ten years [10].

Hospitalization rate for minor LEAs may be underestimated, since interventions done in the outpatients clinical settings are not considered; on the contrary, we can assume that hospitalization rate for major amputations is quite valid as they are usually made in the hospital setting.

Our data show that male sex is at higher risk of LEAs than females, confirming what emerged from previous published studies [4], [16], [18]. Advanced age plays a very significant role in the incidence of LEAs, and males present a much higher age-related ratio of minor to major amputation rate, in agreement with previously reported data [30].

A limitation of our study, as of all the studies based on administrative databases, is the quality of data, and in particular the possible underreporting of diabetes diagnosis. It's known that retrospective methods may underestimate amputation rates [31] and, in addition, our data are collected for administrative purposes, and under-reporting of diabetes is possible when not related to specific costs. We were able to verify our data only through an internal control, concerning repeated discharges for the same patient along the whole study period showing a 10% underestimation. Nevertheless, even if we cannot exactly evaluate the accuracy in reporting diabetes in the discharge records, local experience, as well as widely previously published information, demonstrate that hospital discharge can be considered an efficient and inexpensive way for estimating amputation rates among persons with diabetes [32], [33].

Moreover, the quality of registration has improved from 1994, year in which the National database was established, and is currently regarded as highly complete (http://www.archeo.salute.gov.it/ricoveriOspedalieri/ricoveriOspedalieri.jsp). On the other hand, it must be outlined that the strength of our data is linked to the use of an official database, covering all the Italian population for a long observation period (2001–2010).

In conclusion main results of this study suggest that major amputation rate is progressively declining in Italy in the ten-year-period 2001–2010, probably due to the improvement in quality of care of diabetic foot during these years. This result appears even more important since it has been accomplished in presence of rising prevalence of diabetes.

## Supporting Information

Table S1 **Crude (and age/sex standardized) amputee rate and hospitalization rate for amputation (per 100,000 residents) in people with diabetes. Italy, 2001–2010.**
(DOC)
