# Supplementary material for: Lower Extremity Amputations in Persons with and without Diabetes in Italy: 2001–2010
Source: PLoS One. 2014 Jan 28;9(1):e86405. doi: 10.1371/journal.pone.0086405 (PMC3904875; doi:10.1371/journal.pone.0086405)
Supplement: Table S1 — Crude (and age/sex standardized) amputee rate and hospitalization rate for amputation (per 100,000 residents) in people with diabetes. Italy, 2001–2010. (DOC) [file pone.0086405.s001.doc]

|  | **People with diabetes** | | | | | |
| --- | --- | --- | --- | --- | --- | --- |
|  | **Minor** | | **Major** | | **Total** | |
|  | Amputee rate | Hosp.  Rate | Amputee  rate | Hosp.  rate | Amputee  rate | Hosp.  rate |
| **2001** | na | 7.1 (7.1) | na | 4.3 (4.3) | na | 12.0 (12.0) |
| **2002** | na | 8.2 (8.2) | na | 4.9 (4.9) | na | 13.7 (13.7) |
| **2003** | 6.5 (6.5) | 8.5 (8.4) | 4.4 (4.3) | 4.7 (4.6) | 11.5 (11.3) | 13.9 (13.7) |
| **2004** | 7.2 (7.0) | 9.3 (9.1) | 4.3 (4.1) | 4.5 (4.3) | 11.9 (11.6) | 14.3 (14.0) |
| **2005** | 7.4 (7.1) | 9.5 (9.2) | 4.2 (4.0) | 4.5 (4.3) | 12.0 (11.6) | 14.5 (14.0) |
| **2006** | 7.4 (7.1) | 9.5 (9.1) | 4.2 (4.0) | 4.4 (4.2) | 12.1 (11.5) | 14.5 (13.9) |
| **2007** | 7.2 (6.8) | 9.1 (8.7) | 3.9 (3.6) | 4.1 (3.8) | 11.5 (10.8) | 13.7 (12.9) |
| **2008** | 7.5 (7.1) | 9.6 (9.0) | 4.2 (3.9) | 4.5 (4.2) | 12.1 (11.3) | 14.7 (13.7) |
| **2009** | 7.9 (7.4) | 10.0 (9.3) | 4.0 (3.6) | 4.2 (3.8) | 12.3 (11.4) | 14.7 (13.6) |
| **2010** | 7.9 (7.3) | 10.1 (9.3) | 3.9 (3.5) | 4.1 (3.7) | 12.2 (11.2) | 14.7 (13.4) |

Standard: 2001 Italian resident population
